# Supplementary material for: Multiomics biomarkers were not superior to clinical variables for pan-cancer screening
Source: Commun Med (Lond). 2024 Nov 17;4:234. doi: 10.1038/s43856-024-00671-z (PMC11570627; doi:10.1038/s43856-024-00671-z)
Supplement: Supplementary file 2 — Supplementary Information [file 43856_2024_671_MOESM2_ESM.pdf]

Supplementary Information

Supplementary Methods

Data source and study participants

The UK Biobank (UKBB) study is a prospective cohort study comprising more than 500,000 individuals recruited in the United Kingdom[1]. Ethical approval for the UKBB was obtained from the National Information Governance Board for Health and Social Care and the National Health Service Northwest Multi-Center Research Ethics Committee[1]. Prior to enrollment, all participants provided informed consent electronically. This research was conducted in compliance with the approved UKBB Project ID 102162.

Nuclear magnetic resonance (NMR) spectroscopy was conducted in two phases: Phase 1 (June 2019 to April 2020) and Phase 2 (April 2020 to June 2022) using eight spectrometers at Nightingale Health in Finland. The NMR dataset, comprising 249 metabolite measurements, covered lipoprotein lipids in 14 subclasses, fatty acids, amino acids, ketone bodies, and glycolysis metabolites, quantified in molar concentration units. This dataset, along with its associated quality control (QC) matrices, was processed using the ukbnmr package (version 2.0)[2]. Of the 249 measurements, 168 were absolute, and 81 were ratios. Preprocessing involved technical variation removal and computation of an additional 76 biomarkers from the post-QC dataset. The updated version 2 of the ukbnmr V2 package was used to eliminate technical variations, considering the well positions within each batch separately. A total of 325 metabolite measurements were retained for subsequent analyses, excluding repeat assessments for individuals with both baseline (2006-2010) and repeat (2012-2013) data. Technical variation removal was performed using the ukbnmr V2 package, and further details on this process are available in the ukbnmr GitHub repository.

Proteomic profiling of blood plasma samples was carried out between 2006 and 2010 using the Olink Explore 1536 platform, which measures 2,941 protein analytes that capture 2,923 unique proteins. The inclusion criteria and specifics of the proteomics assays and normalization processes used are outlined in a prior study[3].

Genotyping, imputation, and quality control procedures for the UKBB genomic data were performed as previously described. Genome-wide data in the BGEN v1.2 format from the

UKBB v3 imputed data were utilized. All NMR, proteomic, and genomic data were processed using specific codes (3,143, and 87), facilitating data decoding.

Diagnosis Assessment and Patient Group Identification

To identify cancer cases, the earliest reported data across the respective columns for the date of first inpatient diagnosis (field 40005) from the UKBB were considered. A group of healthy controls was identified, consisting of patients without any disease code (fields 40006, 40013, 41201, 41202, 41203, 41204, 41205, 41270, and 41271). Similarly, a group of non-cancer controls was identified, consisting of patients without any cancer code (field 40005). The disease codes were evaluated with respect to the end of the follow up period, which means that if patient didn't have and cancer code (field 40005), the patient was not diagnosed with the cancer until the 31st of October 2022. The matching process utilized the nearest neighbor method with Euclidean distance as a measure of similarity. Exact matching for sex was implemented in the matching procedure. Individuals who decided to withdraw from the UKBB were removed from the analysis.

Weighted polygenic risk score (PRS) analyses

The genetic variants used in this study were genetic variants that are published in the cancer-specific polygenic risk score reported in the PGS catalog[4]. The cancers included were breast (PGS000072), prostate (PGS000084), colorectal (PGS000074), malignant melanoma (PGS000079), non-Hodgkin lymphoma (PGS000080), cervical (PGS000073), ovarian (PGS000082), bladder (PGS000071), leukemia (PGS000077), kidney (PGS000076), thyroid (PGS000087), and lung (PGS000078) diseases. First step, for each cancer specific PRS, each reported genetic variant was weighed by its reported effect size and used to calculate the individual risk score for this specific cancer. For the combined PRS, the genetic variants reported to be associated with each cancer (same genetic variants used in the first step) were grouped together and used to calculate the individual combined risk score. There was no selection of genetic variants based on functional consequence or based on the genes these genetic variants were mapped to. In the above-mentioned steps, genetic variants (SNPs) were pruned and weighted following the standard procedures.

Modeling the probability of health status

K-nearest neighbor imputation was specifically performed using the KNNImputer method[5], which was chosen for its computational efficiency, simplicity, and comparable performance to more complex methods, particularly in datasets with a low prevalence of missing values[6].

We used the get_dummies function and MinMaxScaler function from the sklearn package to preprocess the clinical variables. Proteomics and Metabolomics data were preprocessed by UKBB and CPTAC respectively.

A total of 10 000 trees were analyzed and used in the extremely randomized trees (ERT) method to reduce the impact of multicollinearity.

Cross-validation, implemented in the LogisticRegressionCV method from the sklearn package, was used to optimize the L2 penalty factor. We used the N most important variables based on the ERT method as features and binary disease status as the response variable. Despite the potential slight reduction in model performance, logistic regression with the L2 penalty was preferred because of its interpretability, which is crucial in clinical research. Logistic regression also facilitates the assessment of each feature's positive or negative association with the disease, aligning with the interpretative needs of clinicians familiar with this method.

The overall pipeline performance was assessed by applying the trained imputation model, downsizing the feature set based on the ERT model, and utilizing the trained logistic model on the test datasets. The AUCs were computed using the roc_auc_score function from sklearn package.

We used the ggpubr package[7] to perform two-sided t tests with false discovery rate adjustment. The false discovery adjustment was performed using the same package using and its default, Holm method.

The analyses were conducted in R (v4.1.1) and Python (v3.7.9), employing default parameters and a random seed of 42 for all analyses unless otherwise specified.

Construction of the interactive atlas

The atlas predicts a set of biomarkers based on the specified settings, with clinical variables and three omics layers available for user selection. To evaluate discriminative performance, receiver operating characteristic (ROC) curves for the test dataset were constructed for the app. When the baseline comparison group was configured as "healthy controls", the atlas

generated ROC curves for prevalent cases versus healthy controls across various molecule types. Similarly, in the "non-cancer" setting, the app produces a test set using patients with diagnoses other than cancer as baseline cases. Users have the flexibility to choose from one to fifteen biomarkers. This Shiny app provides the exploration and interpretation of results, including an interactive and intuitive platform for users to navigate through all the models and visualize the discriminative performance of the predictive models.

Supplementary Results

The gene ontology enrichment of the cancer related proteins

To strengthen the findings and provide biological relevance, we performed enrichment analysis of 15 most disease relevant proteins. For each cancer, we selected the five most significant biological processes based on gene ontology (GO) database and constructed a heatmap to visualize the results (Supplementary Figure 2). We can see that each set of proteins specific for the given cancer has different terms associated with them, most of them with high relevance to the given cancer. For all analyzed cancers together, regulation of cell population proliferation and of different immune cells were shared mechanisms. For individual cancers, more specific branches of these mechanisms were enriched. For example, in colorectal cancer regulation of phagocytosis, NK cell cytotoxicity, endothelial cell migration, serine/threonin signaling and cell proliferation pathways were enriched. These findings agree with the involvement of complex and heterogenous pathways across multiple cell types, and not only tumor cells. The list of all significant terms (adj. p value < 0.05) can be found in Supplementary Data 8.

Supplementary Figure 1. A violin plot showing the probability of developing cancer. The line depicts the median value. * p<0.05, ** p <0.01, *** p < 0.001, **** p<0.0001. T test, adjusted p values

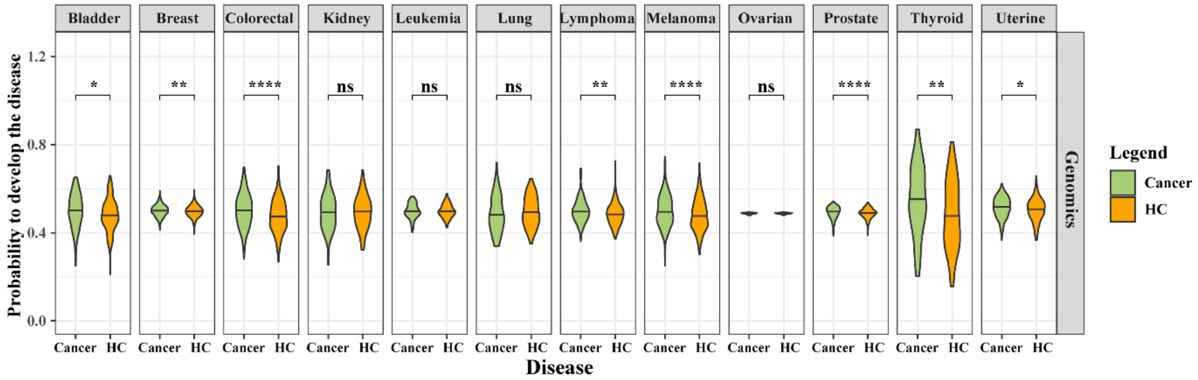

Supplementary Figure 2. A heatmap showing the GO enrichment of cancer specific proteins. The color depicts the adjusted p values in the logarithmic scale.

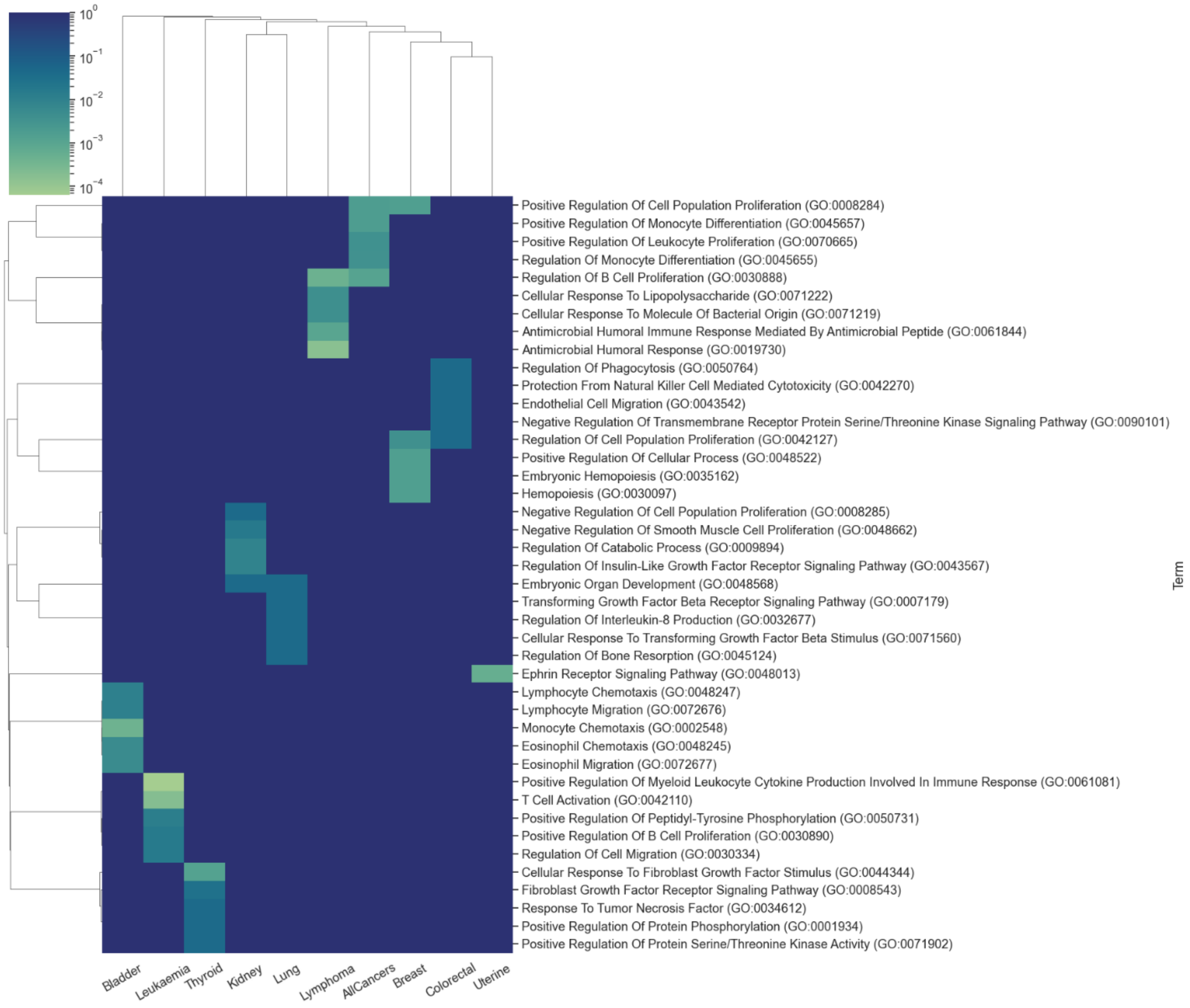

Supplementary References

1. Sudlow C, Gallacher J, Allen N, et al. UK biobank: an open access resource for identifying the causes of a wide range of complex diseases of middle and old age. *PLoS Med*. Mar 2015;12(3):e1001779. doi:10.1371/journal.pmed.1001779
2. Ritchie SC, Surendran P, Karthikeyan S, et al. Quality control and removal of technical variation of NMR metabolic biomarker data in ~120,000 UK Biobank participants. *Sci Data*. Jan 31 2023;10(1):64. doi:10.1038/s41597-023-01949-y
3. Sun BB, Chiou J, Traylor M, et al. Plasma proteomic associations with genetics and health in the UK Biobank. *Nature*. Oct 2023;622(7982):329-338. doi:10.1038/s41586-023-06592-6
4. Gustafsson M, Nestor CE, Zhang H, et al. Modules, networks and systems medicine for understanding disease and aiding diagnosis. *Genome Med*. 2014;6(10):82. doi:10.1186/s13073-014-0082-6
5. Troyanskaya O, Cantor M, Sherlock G, et al. Missing value estimation methods for DNA microarrays. *Bioinformatics*. Jun 2001;17(6):520-5. doi:10.1093/bioinformatics/17.6.520
6. Ge Y, Li Z, Zhang J. A simulation study on missing data imputation for dichotomous variables using statistical and machine learning methods. *Sci Rep*. Jun 9 2023;13(1):9432. doi:10.1038/s41598-023-36509-2
7. Kassambara A. ggpubr: 'ggplot2' Based Publication Ready Plots. https://rpkgs.datanovia.com/ggpubr/
