# Supplementary material for: Multiomics biomarkers were not superior to clinical variables for pan-cancer screening
Source: Commun Med (Lond). 2024 Nov 17;4:234. doi: 10.1038/s43856-024-00671-z (PMC11570627; doi:10.1038/s43856-024-00671-z)
Supplement: Supplementary file 3 — Description of Additional Supplementary Files [file 43856_2024_671_MOESM3_ESM.pdf]

Description of Additional Supplementary Files

Supplementary Data 1. Summary of cancer patients for collective analysis

Supplementary Data 2. Clinical variables used from UKBB

Supplementary Data 3. Summary of data from UKBB

Supplementary Data 4. Summary of the results from UKBB for proteomics (prot) metabolomics (met)

Supplementary Data 5. Summary of UKBB results for Genomics

Supplementary Data 6. Summary of the participants from CPTAC study

Supplementary Data 7. Results summary for CPTAC data

Supplementary Data 8. A list of significantly enriched gene ontology terms
