# Supplementary material for: Multiomics biomarkers were not superior to clinical variables for pan-cancer screening
Source: Commun Med (Lond). 2024 Nov 17;4:234. doi: 10.1038/s43856-024-00671-z (PMC11570627; doi:10.1038/s43856-024-00671-z)
Supplement: Supplementary file 12 — Reporting Summary [file 43856_2024_671_MOESM12_ESM.pdf]

nature portfolio

Corresponding author(s): Mikael Benson

Last updated by author(s): Mar 25, 2024

Reporting Summary

Nature Portfolio wishes to improve the reproducibility of the work that we publish. This form provides structure for consistency and transparency in reporting. For further information on Nature Portfolio policies, see our Editorial Policies and the Editorial Policy Checklist.

Statistics

For all statistical analyses, confirm that the following items are present in the figure legend, table legend, main text, or Methods section.

n/a	Confirmed	
☐	☒	The exact sample size (*n*) for each experimental group/condition, given as a discrete number and unit of measurement
☐	☒	A statement on whether measurements were taken from distinct samples or whether the same sample was measured repeatedly
☐	☒	The statistical test(s) used AND whether they are one- or two-sided<br>*Only common tests should be described solely by name; describe more complex techniques in the Methods section.*
☒	☐	A description of all covariates tested
☐	☒	A description of any assumptions or corrections, such as tests of normality and adjustment for multiple comparisons
☐	☒	A full description of the statistical parameters including central tendency (e.g. means) or other basic estimates (e.g. regression coefficient) AND variation (e.g. standard deviation) or associated estimates of uncertainty (e.g. confidence intervals)
☐	☒	For null hypothesis testing, the test statistic (e.g. *F*, *t*, *r*) with confidence intervals, effect sizes, degrees of freedom and *P* value noted<br>*Give P values as exact values whenever suitable.*
☒	☐	For Bayesian analysis, information on the choice of priors and Markov chain Monte Carlo settings
☒	☐	For hierarchical and complex designs, identification of the appropriate level for tests and full reporting of outcomes
☒	☐	Estimates of effect sizes (e.g. Cohen's *d*, Pearson's *r*), indicating how they were calculated

Our web collection on statistics for biologists contains articles on many of the points above.

Software and code

Policy information about availability of computer code

Data collection

The multiomics and clinical data was collected from UKBiobank(application number 102162) and Clinical Proteomic Tumor Analysis Consortium (CPTAC)

Data analysis

We used various open-source R and Python packages in our study. The methods and specific functions employed are described in detail in the methodology section, while the corresponding computer code is accessible on the GitHub open-source repository. A link to the data analysis pipeline for data reproducibility is also included in the manuscript. Some of the tools and methods utilized include the ukbtools package (https://cran.r-project.org/web/packages/ukbtools/index.html), logistic regression, extremely randomized trees and sklearn KNNImputer. The analysis were conducted in R (v4.1.1) and Python (v3.7.9). We used default parameters in the methods and a random seed of 42 for all analyses unless otherwise specified.

For manuscripts utilizing custom algorithms or software that are central to the research but not yet described in published literature, software must be made available to editors and reviewers. We strongly encourage code deposition in a community repository (e.g. GitHub). See the Nature Portfolio guidelines for submitting code & software for further information.

Data

Policy information about availability of data

All manuscripts must include a data availability statement. This statement should provide the following information, where applicable:

- Accession codes, unique identifiers, or web links for publicly available datasets
- A description of any restrictions on data availability
- For clinical datasets or third party data, please ensure that the statement adheres to our policy

An interactive, web-based atlas for translational researchers to find optimal biomarkers is available at https://macd.shinyapps.io/CancerAtlas/. All data used in this study are available to access from the UK Biobank at https://www.ukbiobank.ac.uk/ for approved researchers through the UK Biobank data-access protocol.

Research involving human participants, their data, or biological material

Policy information about studies with human participants or human data. See also policy information about sex, gender (identity/presentation), and sexual orientation and race, ethnicity and racism.

Reporting on sex and gender	We used self-reported sex (biological attribute) in our study. We aim to develop a strategy to identify and prioritise mechanisms and biomarkers that were consistent across cancers, even if they were sex specific such as ovarian and prostate cancer. Therefore, sex and gender analyses were not performed. However, we considered age and sex as covariates when matching controls to cancer patients.
Reporting on race, ethnicity, or other socially relevant groupings	We identify populations using self reported data provided by the UK Biobank.
Population characteristics	Population characteristics of samples from CPTAC, and UK biobank are described in the respective databases. For example summary characteristics for cancer cases in UK Biobank is provided here: https://biobank.ndph.ox.ac.uk/~bbdatan/CancerSummaryReport.html
Recruitment	The UK Biobank (UKBB) is a prospective population-based research resource focusing on the role of genetic, environmental and lifestyle factors in health outcomes in middle age and later life. More than 9,000,000 men and women between 40 and 69 registered with the UK NHS were invited to take part. Of those, approximately 53,000 UK Biobank participants were randomly selected for proteomics in The Pharma Proteomics Project.
Ethics oversight	UK Biobank has approval from the Northwest Multi-centre Research Ethics Committee (MREC) as a Research Tissue Bank (RTB) approval. This approval means that researchers do not require separate ethical clearance and can operate under the RTB approval (there are certain exceptions to this which are set out in the Access Procedures, such as re-contact applications).

Note that full information on the approval of the study protocol must also be provided in the manuscript.

Field-specific reporting

Please select the one below that is the best fit for your research. If you are not sure, read the appropriate sections before making your selection.

☒ Life sciences ☐ Behavioural & social sciences ☐ Ecological, evolutionary & environmental sciences

For a reference copy of the document with all sections, see nature.com/documents/nr-reporting-summary-flat.pdf

Life sciences study design

All studies must disclose on these points even when the disclosure is negative.

Sample size	Sample size are indicated in the manuscript for each of the different datasets.
Data exclusions	As described in the methods, we excluded cancer types with less than 40 available control samples. We also excluded patients that decided to drop out from the UKBB study
Replication	A machine learning pipeline consisting of data cleaning, data imputation, feature selection, and model training using cross-validation and comparison of the results on holdout test sets
Randomization	N/A
Blinding	N/A

Reporting for specific materials, systems and methods

We require information from authors about some types of materials, experimental systems and methods used in many studies. Here, indicate whether each material, system or method listed is relevant to your study. If you are not sure if a list item applies to your research, read the appropriate section before selecting a response.

Materials & experimental systems

n/a	Involved in the study
☒	☐ Antibodies
☒	☐ Eukaryotic cell lines
☒	☐ Palaeontology and archaeology
☒	☐ Animals and other organisms
☒	☐ Clinical data
☒	☐ Dual use research of concern
☒	☐ Plants

Methods

n/a	Involved in the study
☒	☐ ChIP-seq
☒	☐ Flow cytometry
☒	☐ MRI-based neuroimaging
